# The effect of the COVID-19 pandemic on routine childhood immunization coverage and timeliness in India: retrospective analysis of the National Family Health Survey of 2019–2021 data

**DOI:** 10.1016/j.lansea.2022.100099

**Published:** 2022-10-21

**Authors:** Amit Summan, Arindam Nandi, Anita Shet, Ramanan Laxminarayan

**Affiliations:** a One Health Trust, Washington, DC, USA; b The Population Council, 1 Dag Hammarskjold Plaza, New York, NY, 10017, USA; c International Vaccine Access Center, Johns Hopkins Bloomberg School of Public Health, Baltimore, MD, USA; d One Health Trust, New Delhi, India; e High Meadows Environmental Institute, Princeton University, NJ, USA

**Keywords:** India, COVID-19, Child immunization, SARS-CoV-2, Vaccine, pandemic

## Abstract

**Background:**

The COVID-19 pandemic has disrupted health systems globally. We estimated the effect of the pandemic on the coverage and timeliness of routine childhood immunization in India through April 2021.

**Methods:**

We used data from India's National Family Health Survey 2019–2021 (NFHS-5), a cross-sectional survey which collected immunization information of under-five children from a nationally representative sample of households between June 2019 and April 2021. We used a mother fixed-effects regression model – accounting for secular trends and confounding factors – to compare COVID-affected children with their COVID-unaffected siblings (n = 59,144). Children who were eligible for a vaccine after January 30, 2020 (date of the first COVID case in India) were considered as the COVID-affected group and those eligible for a vaccine before this date were included in the COVID-unaffected group. Coverage of the following vaccine doses was considered—Bacillus Calmette–Guérin (BCG), hepatitis B birth dose (hepB0), DPT1 (diphtheria, pertussis, and tetanus, first dose), DPT2, DPT3, polio1, polio2, polio3, and measles first dose (MCV1). Indicators of vaccine coverage and vaccine timeliness (defined as receiving a dose within 45 days of minimum eligibility age) were separately examined.

**Findings:**

Immunization coverage was lower in COVID-affected children as compared with unaffected children, ranging from 2% lower for BCG and hepB0 to 9% for DPT3 and 10% for polio3. There was no significant difference in MCV1 coverage. Coverage reduction was greater for vaccines doses given in later age groups. The rate of timely receipt of polio and DPT vaccine doses was 3%–5% lower among COVID-affected children relative to unaffected children. Among population subgroups, COVID-affected male children and those from rural areas experienced the highest reduction in vaccine coverage.

**Interpretation:**

Children in India experienced lower routine immunization coverage and greater delays in immunization during the COVID-19 pandemic.

**Funding:**

The 10.13039/100000865Bill & Melinda Gates Foundation.

**Research in context**

**Evidence before the study**

Healthcare seeking and use broadly decreased during the COVID-19 pandemic. We searched PubMed and Google Scholar on June 13, 2022 for articles published after 2020 for the following title keywords: “(COVID OR COVID-19 OR coronavirus)” AND “(child OR children OR infant)” AND “(vaccination OR immunization OR immunisation)”. We focused on articles that included analysis on India and identified two global studies that used administrative dose data and reported a decrease in a select number of vaccines during 2020. A third study that conducted a telephone survey in Rajasthan, India, also found decreased vaccination rates in children in 2020.

**Added value of this study**

This is the first analysis of childhood immunization coverage that uses a nationally representative childhood health survey and a robust modelling framework to control for confounding factors. Administrative dose data, especially from low- and middle-income countries, can suffer from quality issues. This analysis also includes data from the first quarter of 2021, whereas previous analyses focused on 2020.

**Implications of all the available evidence**

Childhood immunization coverage in India decreased markedly during COVID-19. Immunization decreases were greater for vaccines received later in the immunization schedule and for rural households. During the pandemic period, the degree of delay for certain vaccine doses such as polio and DTP vaccines was greater, indicating that more children were unable to get their due vaccines on time. Without appropriate catch-up vaccination efforts, preventable child morbidity and mortality can increase substantially in future years, especially for vulnerable populations.

## Introduction

The COVID-19 pandemic has adversely affected healthcare access around the world, with 15 million excess deaths attributed to its direct and indirect effects.^1^ Over 90% of countries that provide health systems information to the World Health Organization (WHO) were reporting disruptions to essential healthcare programs at the end of 2021, and the proportion of countries reporting disruptions in routine immunization programs increased from 33% to almost 50% between the first and fourth quarters of 2021.^2^

Childhood immunization prevents the spread of infectious diseases, reducing associated morbidity and mortality. By preventing disease episodes during the first 1000 days of life – a crucial developmental phase for young children – immunization can also improve cognitive, education, and economic outcomes in later life.^3^, ^4^, ^5^, ^6^ However, an estimated 23 million children did not receive DPT3 (diphtheria, pertussis, and tetanus vaccine, third dose) in 2020–3.7 million more than in 2019^7^—and 60% of these children lived in 10 low- and middle-income countries (LMICs) including India.^8^ An analysis of 170 countries using administrative vaccination data found reduced coverage of DPT3 and measles-containing vaccine first dose (MCV1) in the first half of 2020 with a rebound in the latter half.^9^ In the WHO South-East Asia region, administered DPT3 doses dropped by 57% in April 2020 compared with April 2019.

India had among the largest reductions in childhood vaccination coverage. The WHO estimated that DPT3 vaccination rates in India fell from 91% in 2019 to 85% in 2020.^7^ A study using administered dose data similarly estimated that DPT3 vaccination dropped 15.8% in 2020 in India, relative to the previously projected vaccination rate.^10^ Another study from Rajasthan conducted phone interviews for 2,144 children between January and October 2020, and found that children in heavily COVID-19–exposed areas were less than half as likely to get vaccinated by nine months of age, relative to unexposed children.^11^

These studies have limitations inherent in modelled and administrative data because they tend to present aggregate statistics without accounting for potential confounding factors, including underlying secular trends in coverage and differences in individual and household-level factors that influence vaccinations, such as age, sex, parental education, beliefs, and access to healthcare facilities. Additionally, the quality of administrative data, particularly at the subnational level, is limited as compared with in-depth retrospective household surveys. Lastly, these studies focused on only DPT3 and MCV vaccinations and did not address the coverage of other vaccines or the timely receipt of doses. Considering that DPT3 coverage rate in India increased rapidly from 78% in 2015–2016 to 91% in 2019^7^^,^^12^ – aided by special immunization drives^13^^,^^14^ – it is important to understand and quantify the backsliding in progress due to the pandemic.

Aiming to estimate the effect of the COVID-19 pandemic on routine childhood immunization coverage and timeliness in India, we examined the status of standard routine childhood vaccines given in the first year of life in India using data from a large, nationally representative household survey.

## Methods

### Data

We used data from the fifth round of the National Family Health Survey (NFHS-5), conducted between June 2019 and April 2021.^15^ The survey was conducted in two phases. Phase 1 extended from June 2019 to January 2020, covering 22 states and union territories; phase 2 was from January 2020 to April 2021, covering the remaining 14 states and union territories. NFHS-5 covered 636,699 households in 707 districts across all 36 jurisdictions, and it included 232,920 children under the age of five years. NFHS-5 was a stratified two-stage sample survey, where the 2011 census was used for the sampling frame for primary survey units (PSUs). In rural areas, PSUs were villages and in urban areas, were Census Enumeration Blocks. The survey collected immunization data on all children born after 2016, including vaccine dose and receipt date, from vaccination cards or from maternal recall when a card was unavailable. We included all 232,920 under-five children from both phases of the survey in our analysis. Of these, children from phase 2 states who had at least one sibling (n = 59,144) contributed to the variation in outcomes, as discussed in the next section.

We examined nine vaccine doses that are recommended during the first year of life: Bacillus Calmette–Guérin (BCG), hepatitis B birth dose (hepB0), DPT1 (diphtheria, pertussis, and tetanus, first dose), DPT2, DPT3, polio1 (polio, first dose), polio2, polio3, and first dose of measles-containing vaccine (MCV1).^16^ The recommended age for administering MCV1 is nine to 12 months; all the other vaccines are given within 14 weeks following birth. Newer vaccines (rotavirus vaccine and pneumococcal conjugate vaccines) were not included. We analyzed three sets of outcome variables. The first is a binary coverage indicator: (i) receipt of a vaccine dose vs. non-receipt. The two others are delay indicators: (ii) receipt of a vaccine dose within 28 of the minimum eligibility age vs. receipt after 28 days of minimum eligibility or non-receipt, and (iii) receipt of a vaccine dose within 45 days of the minimum eligibility vs. receipt after 45 days of minimum eligibility or non-receipt. Previous studies have used similar 28-day and 45-day definitions of delay to assess vaccination timeliness.^13^^,^^17^^,^^18^

We excluded children with implausible data, such as having been immunized prior to their birth date. Additionally, if vaccination of a higher dose was reported but previous doses were marked as not received, we changed the value of the higher-dose vaccine to missing; this accounts for 0.14% of observations. For example, if DPT3 was reported as being received but the child did not receive DPT1, the child would receive a missing value of DPT3 to account for measurement errors. For the delay indicators, we excluded children for whom vaccination receipt was reported more than one week prior to their minimum eligibility age.

### Empirical approach

We employed a linear probability model (LPM) with mother fixed-effects to identify the differences in vaccination between siblings born before and after the COVID-19 pandemic. Household or mother fixed-effects have commonly been used in public health studies to assess the variation in health outcomes between siblings of different sex or age.^19^^,^^20^ In our data, background characteristics of children—such as household standard of living; demographic, health, and educational indicators of parents; and community-level access to healthcare and quality of service delivery, including immunization—can systematically differ between the groups affectsed or unaffected by COVID. If these differences are correlated with immunization outcomes, simple group differences in the outcomes indicator or least squares-based regression results of the relationship between COVID-19 exposure and immunization outcomes would yield biased estimates. To mitigate such biases, we included mother fixed-effects in our regression model, which accounted for all observed and unobserved characteristics at the level of the mother and above (e.g., household, district, and state). The LPM for vaccine *x* can be written as:

$$V_{x,i}=\partial T_{x,i}+\;\beta X_{i}+\gamma M_{i}+\;\varepsilon_{i}$$

where *V*_*x,i*_ denotes the dichotomous outcome indicator (receipt vs. nonreceipt, or timely receipt vs. delayed receipt of vaccine *x*) for child *i*. *T*_*x,i*_ is the binary indicator of COVID-affected or COVID-unaffected group, which is defined based on age of vaccine eligibility as below:

$$T_{x,i}=1\;if\;age_{i}<eligibilit{y_{age}}_{x}+delay_{x}\;,\;and\;0\;otherwise$$

where *age*_i_ is the age of child *i* at the start of COVID-19 period, and *eligibility*_*agex*_ is the age of eligibility for a child for vaccine *x*, and *delay*_*x*_ is the median delay (days) with which children in India received that vaccine *x* in 2019. The COVID-affected period start date is the date of the first reported COVID-19 case in India, January 30, 2020. For example, consider a child who was 9 weeks old on January 30, 2020, when the first case of COVID-19 was detected. The child was older than the minimum eligibility age for DPT1 (6 weeks) plus the median 2019 delay in DPT1 vaccination (2 weeks) and therefore was assigned to the control (COVID-unaffected) group for DPT1 analysis. In comparison, a child who was 7 weeks old on January 30, 2020 would be assigned to the COVID-affected group since they had not reached the minimum eligibility plus median delay age (6 + 2 = 8 weeks) for DPT1. Our definition of COVID-affected group was based on age and vaccine eligibility and was unrelated to COVID-19 infection or exposure of a child (to another infected individual) for which NFHS-5 did not collect data.

Mother fixed-effects included in the model are denoted by *M*_*i*_ and *X*_*i*_ is a vector of the following sibling level covariates: child's age in months, sex, birth order (first, second, third, fourth, or higher), and a binary indicator of institutional delivery. Similar to previously published mother fixed-effects studies,^19^^,^^21^^,^^22^ the source of variation in the variable of interest comes from children under the age of five years who have at least one sibling (from NFHS-5 phase 2 states). However, to improve the goodness of fit of the model (e.g., the R^2^ statistic), we included all children with and without siblings (survey phases 1 and 2) in our model following previous studies,^19^^,^^21^^,^^22^ even though children without siblings would not help explain the variation in the outcome variable.

We conducted additional analysis by subgroup: rural vs. urban, female vs. male, and low-wealth vs. high-wealth households. High-wealth households were in the top three wealth quintiles, and low-wealth households were in the bottom two wealth quintiles. STATA version 14.2 was used for all analysis. Standard errors were robust and clustered at the mother level and p-value of <0.05 was considered for statistical significance.

### Role of the funding source

The funder had no role in study design, analysis, preparation of the manuscript, or the decision to submit for publication. AS(1) and AN had full access to all the data set, and AN and RL had full responsibility for submitting the paper for publication.

## Results

### Summary statistics

Table 1 presents the difference between the COVID-affected and COVID-unaffected groups for DPT3 coverage and delay and sample characteristics. Summary statistics for other vaccine doses were similar and are not presented separately. There were 18,803 children in the affected group and 214,117 children in the unaffected group (including children without siblings). COVID-unaffected children had higher DPT3 vaccination rates than COVID-affected children (84% vs. 70%, p-value <0.01) and less delay in vaccination based on the 45-day measure (57% vs. 59%, p < 0.01).

**Table 1:** Differences in socioeconomic characteristics between COVID-affected and COVID-unaffected groups for DPT3 vaccination.

	Affected mean	Affected SD	Unaffected mean	Unaffected SD	Difference	P-value
DPT-3	0.70	0.46	0.84	0.37	−0.13∗∗	0.00
DPT-3 delay	0.57	0.49	0.59	0.49	−0.01	0.01
**Region**						
North	0.23	0.42	0.18	0.38	0.05∗∗	0.00
Central	0.50	0.50	0.23	0.42	0.26∗∗	0.00
East	0.16	0.37	0.20	0.40	−0.03∗∗	0.00
Northeast	0.05	0.21	0.16	0.36	−0.11∗∗	0.00
West	0.00	0.04	0.10	0.30	−0.1∗∗	0.00
South	0.06	0.24	0.13	0.34	−0.07∗∗	0.00
**Locality**						
Urban	0.19	0.39	0.20	0.40	−0.01∗∗	0.00
**Religion**						
Hindu	0.81	0.39	0.73	0.45	0.08∗∗	0.00
Muslim	0.11	0.31	0.15	0.36	−0.04∗∗	0.00
Sikh	0.03	0.18	0.09	0.28	−0.05∗∗	0.00
Christian	0.03	0.16	0.02	0.13	0.01∗∗	0.00
Other	0.02	0.15	0.02	0.15	0.00	0.86
**Caste**						
SC	0.23	0.42	0.20	0.40	0.02∗∗	0.00
ST	0.20	0.40	0.20	0.40	0.00	0.13
OBC	0.41	0.49	0.38	0.49	0.03∗∗	0.00
Other	0.16	0.37	0.22	0.41	−0.05∗∗	0.00
**Household size**						
>4	0.78	0.42	0.74	0.44	0.04∗∗	0.00
**Head age**						
<21	0.01	0.09	0.01	0.07	0∗∗	0.00
21–31	0.23	0.42	0.24	0.42	−0.01∗∗	0.00
31–41	0.17	0.37	0.24	0.43	−0.08∗∗	0.00
>41	0.60	0.49	0.51	0.50	0.08∗∗	0.00
**Head sex**						
Female	0.14	0.35	0.15	0.36	−0.01∗∗	0.00
**Marital status**						
Married	0.99	0.08	0.98	0.13	0.01∗∗	0.00
**Mother education**						
No education	0.20	0.40	0.22	0.42	−0.02∗∗	0.00
Primary	0.11	0.32	0.13	0.34	−0.02∗∗	0.00
Secondary	0.51	0.50	0.51	0.50	0.00	0.95
Higher	0.17	0.38	0.13	0.34	0.04∗∗	0.00
**Child sex**						
Female	0.48	0.50	0.48	0.50	0.00	0.38
**Child age (months)**						
<3	0.39	0.49	0.03	0.18	0.36∗∗	0.00
3–6	0.18	0.38	0.04	0.19	0.14∗∗	0.00
6–12	0.29	0.45	0.08	0.26	0.21∗∗	0.00
>12	0.14	0.35	0.85	0.35	−0.72∗∗	0.00
**Birth order**						
1	0.40	0.49	0.38	0.49	0.02∗∗	0.00
2	0.32	0.47	0.33	0.47	−0.01∗∗	0.00
3	0.16	0.36	0.16	0.36	0.00	0.64
>3	0.13	0.33	0.13	0.34	−0.01∗∗	0.00
**Delivery place**						
Institutional	0.89	0.31	0.86	0.35	0.03∗∗	0.00
Sample size	18,803		214,117			

Note: *HepB0*, hepatitis B given at birth; *DPT*, diphtheria, pertussis, tetanus; *BCG*, Bacillus Calmette–Guérin; standard errors are below coefficients. +p < 0.1, ∗p < 0.05, ∗∗p < 0.01. Treatment group comprises children who did not reach eligibility age for DPT3 at time of first COVID-19 lockdown.

For background characteristics indicators, there were geographical differences driven by the timing of the survey in the specific area. The largest difference was the greater proportion of COVID-affected children vs. unaffected-COVID children in the Central region (50% vs. 23%, p < 0.00) and a lower proportion in Northeast region (5% vs. 16%, p < 0.01). Other major differences were a higher number of COVID-affected children from Hindu households and children with institutional delivery. The mean age of children in the affected group was lower than the unaffected group (5.7 months vs. 31.9 months, p < 0.01).

### Regression results: vaccination coverage

Table 2, column A, presents the summary of the mother fixed-effects LPM results presenting the coefficient on the intervention (COVID-affected) variable for each vaccine. Full results are shown in Appendix Table A1. Coverage among COVID-19 affected children were lower than their unaffected siblings who were born before the pandemic (2015–2019) for all vaccines except for MCV1. For doses given at birth, the likelihood of receiving BCG and hepB0 was 2% lower among COVID-affected children as compared with their unaffected counterparts. The decrease in vaccination probability for later doses of DPT and polio was greater than for earlier doses. There was a 7% decrease in probability for DPT1 and polio1 vaccination and a 10% decrease for polio3.

**Table 2:** Summary results of effects of COVID-19 on vaccination outcomes.

Vaccine	Receipt of vaccination (A)			Receipt of vaccination within 45 days of eligibility (B)			Receipt of vaccination within 28 days of eligibility (C)		
	Coefficient∗	N	N (siblings)	Coefficient∗	N	N (siblings)	Coefficient∗	N	N (siblings)
BCG	−1.94∗∗ (0.006)	132,335	59,506	−0.47 (0.01)	113,421	50,121	0.15 (0.011)	113,421	50,121
HepB0	−2.49∗ (0.01)	131,240	58,972	−1.69 (0.011)	115,589	51,620	−1.28 (0.011)	115,589	51,620
DPtD1	−7.06∗∗ (0.009)	127,232	56,885	−5.4∗∗ (0.015)	107,661	47,440	−2.83+ (0.016)	107,661	47,440
DPT2	−7.9∗∗ (0.011)	123,278	54,942	−4.59∗∗ (0.015)	105,649	46,504	−2.3 (0.015)	105,649	46,504
DPT3	−9.27∗∗ (0.012)	120,074	53,317	−3.38∗ (0.015)	104,550	45,883	−2.43+ (0.014)	104,550	45,883
Polio1	−6.98∗∗ (0.009)	127,618	57,063	−4.94∗∗ (0.014)	108,685	47,976	−2.97∗ (0.015)	108,685	47,976
Polio2	−8.21∗∗ (0.011)	123,368	55,011	−5.19∗∗ (0.014)	108,904	48,014	−3.79∗ (0.015)	108,904	48,014
Polio3	−10.28∗∗ (0.012)	120,282	53,433	−3.91∗∗ (0.014)	110,970	48,982	−3.05∗ (0.013)	110,970	48,982
Measles	1.68 (0.017)	99,223	43,513	1.85 (0.023)	82,161	35,443	2.26 (0.022)	82,161	35,443

Note: ∗Coefficient of COVID-affected indicator. *BCG*, Bacillus Calmette–Guérin; *HepB0*, hepatitis B given at birth; *DPT*, diphtheria, pertussis, tetanus; standard errors in parentheses. +p < 0.1, ∗p < 0.05, ∗∗p < 0.01.

### Regression results: delay in vaccination

Table 2, columns B and C, presents the summary of the LPM results presenting the coefficient on the intervention variable for each vaccination delay variable. Full results are presented in Appendix Tables A2 and A3. In the main delay model (vaccination within 45 days of eligibility vs. after 45 days or no vaccination), the probability of timely vaccination among COVID-affected children was lower by 3% for DPT3, 4% for polio3, and 5% for DPT1, DPT2, polio1, and polio2 for as compared with COVID-unaffected children. No significant effect was found for the doses given at birth or for MCV1. For vaccination within 28 days vs. after 28 days or no vaccination, only polio doses had a significant reduction in the probability of timely vaccination for COVID-affected children, ranging from 3% to 4%.

### Subsample analysis

Appendix Table A4 presents the summary results for the subsample analysis for vaccination receipt and delay in vaccination, respectively. For vaccination receipt, results vary across vaccines. Reduction in vaccination coverage rates in the COVID-affected group were highest among male children and those from rural areas, relative to their unaffected counterparts.

For DPT3, for example, there was a decrease in probability of vaccination of 15% vs 8% for rural vs. urban households. For delay in DPT1, polio1, polio2, and polio3 vaccination, COVID-affected children in rural households experienced a greater delay in vaccination than urban households. For DPT1, DPT2, and polio2, COVID-affected children in high-wealth households had a greater delay than unaffected children, relative to COVID-affected children in low-wealth households. Similar to whole sample models, no significant effect was found for birth doses and measles in the delay variables.

## Discussion

The adverse effects of the COVID-19 pandemic are far reaching, and extend beyond the immediate negative health and economic outcomes. We find that children born in India after COVID-19 had 2%–10% lower probability of immunization and 3%–5% lower probability of timely vaccination as compared with their siblings who were born prior to the pandemic. For overall vaccination coverage, vaccines given later in the immunization schedule had greater delay than early-dose vaccines.

There are several potential drivers of these effects. National lockdowns, such as those in India, restricted mobility of individuals, whether they were strongly advised or effectively banned from leaving their homes, or they were unable to access transportation due to reduced services.^23^^,^^24^ Fear of contracting COVID-19 caused people to delay accessing healthcare.^25^ Resources including health workers were diverted from routine care to COVID-19 care, reducing the supply of routine services.^25^ The lockdown caused closure of *Angadwadi* centers (community level mother-child nutrition and welfare centers) across rural India, where children access vaccination services.^24^ Healthcare workers faced several challenges in effectively delivering care due to fear of infection, stress, lack of transportation, inadequate protective equipment, and community resistance and stigma.^26^

For timely vaccination, there was no significant difference between COVID-affected and COVID-unaffected children for doses given at birth. These results may be due to the gradual increase in institutional delivery rates across children—from 83% of children born in 2015 to 89% of children born in 2020. Infants born in institutional facilities have direct access to birth dose vaccines and more antenatal care interactions, which can increase demand for vaccination. For later-dose vaccines, however, households must make a separate trip to a health center or immunization session site.

Our estimates support findings from studies that have used administered dose data. Two global studies^9^^,^^10^ found substantial decreases in routine child immunization; these findings were confirmed in many LMIC country specific studies which used varying data sources including in Pakistan,^27^ Equador,^28^ and the sub-Saharan Africa region.^29^ The effect of the COVID-19 pandemic on child vaccination mirrors the experience of many countries in the west Africa region during the Ebola outbreak, where basic vaccination coverage dropped.^30^^,^^31^ These lower vaccination rates were linked to increased measles incidence and measles outbreaks in affected countries post-Ebola.^32^

A previous modelling study estimated a decrease in DPT3 vaccination of 16% in 2020 in India relative to what rates may have been without COVID-19.^10^ A second global study^9^ found that DPT3 doses decreased by 57% in the South-East Asia region. Our estimates suggest a 9% decrease in the DPT3 vaccination rate of children born after the pandemic. The smaller magnitude decrease may be driven by a potential recovery as our study includes children sampled between January and April 2021 or may be driven by our empirical approach. The previous studies^9^^,^^10^ used administered dose data to estimate the effects of COVID-19 on vaccination, while we used household survey data. Second, our methodological approach employed a robust modelling framework with the mother-fixed effects model to account for potential confounding factors.

These results have important implications for India's immunization program and COVID-19 recovery policies. The indirect effects from the pandemic will become apparent only over the next several years. Reduced vaccination coverage will substantially increase the already large morbidity and mortality burden in children. Vaccine-preventable diseases (VPDs), including pneumonia, diarrheal diseases, measles, and meningitis, were responsible for an estimated 400,000 under-five deaths in India in 2015.^33^ Increased delay in vaccine receipt has additional implications for morbidity and mortality, especially for highly contagious diseases such as measles which can retard long-term immunity against other diseases.^34^^,^^35^ A child infected with measles may infect 12–18 other individuals, and an episode of measles can reduce children's innate immunity by a period of up to 2 years, which together can rapidly increase the burden of VPDs in a community.^34^^,^^36^, ^37^, ^38^ A study of 45 LMICs found that only in an estimated 25% of the countries, children were vaccinated close to their vaccination schedule.^39^ In India, 35% of measles vaccine doses given to 10–23 month old children in 2016 were late by four weeks or more.^40^ Timely vaccination is an often ignored but integral component of a successful immunization program.

An estimated 32 million children were born in India between February 2020 and April 2021.^41^ Assuming an 89.5% pre-pandemic DPT3 vaccination rate and a 9% lower probability of vaccination in the exposed population, we calculate that more than 2.5 million doses of DPT3 were missed during this COVID-19 period. In recent years, India's Mission Indradhanush (MI) and Intensified Mission Indradhanush (IMI) campaigns have improved coverage and reduced delays in vaccination.^13^^,^^14^ Future iterations of IMI and other campaigns need to consider the additional resources required to immunize the millions of children missed during the pandemic. We found that children in rural areas may have suffered the greatest decrease in vaccination rates. Funding for catch-up vaccination will need to be in addition to the estimated more than $560 million gap that already exists to reach 90% vaccination rates.^42^ Health facilities should have enough capacity to handle routine health services; research has shown that prior to the pandemic, health facility quality was significantly associated with child vaccination outcomes in India.^18^

It will also be important to evaluate pandemic preparedness and response policies in terms of their broad costs and benefits. A 2020 modelling analysis found that continued routine immunization service provision in Africa would result in greater deaths averted than the deaths caused by increased Ebola transmission from these visits.^43^ Similar analysis is needed for other regions and throughout a pandemic as virus strains evolve or individuals get protection through vaccination. Broadly, lockdowns and pandemic policy should encourage and allow safe access to routine immunization services.

Our analysis has important limitations. First, our identification strategy only allows for the comparison of outcomes between siblings — therefore, we do not capture the change in vaccination from children that did not have a sibling, even though they may have experienced a decrease in vaccination. However, the mother fixed-effects model allows for robust estimates of the effect of COVID-19 on vaccination status by examining siblings that would share all household level characteristics. Second, not all children had complete vaccination cards, and data based on the mother's recollections can be subject to recall error. To check for potential bias, we ran our main models only for children with vaccination cards, but the results remained largely unchanged. Third, it is possible that some children who became eligible for a vaccine close to the pandemic start date were assigned to the control group in error. This might happen for children with larger-than-median delay in vaccination. For example, consider a child nine weeks old at pandemic start date. They have passed the minimum eligibility date for DPT1 plus the median delay period for DPT1 of two weeks and are therefore assigned to the control group. However, if this household typically has a delay of four weeks in vaccinations, the child may have been affected by COVID-19 and should be in the treatment group. Such children constitute only a very small portion of our sample, but the effect of their misassignment would be to decrease the vaccination rate and increase the delay of COVID-affected children, essentially decreasing the coefficient of interest. Finally, we excluded vaccines which were recently introduced in the national program, such as the pneumococcal and rotavirus vaccines, as they were not available nationally by 2019.^44^

Child immunization is one of the most cost-effective health interventions. Missed vaccinations will increase preventable child mortality and morbidity and have important secondary effects—poorer cognitive, education, and economic outcomes for children in later life. Future immunization resources in India must consider the additional cost of catch-up vaccination for children who have missed doses. Future pandemic response and preparedness policies must ensure that routine health services, including child immunization services, remain robust during infectious disease outbreaks.

## Contributors

AS(1) and AN designed the study. AS(1) conducted the analysis and wrote the first version of the manuscript. AS(1) and AN had access to, and have verified, the data. AN, RL, and AS(2) interpreted the findings and critically evaluated and edited the manuscript. All authors approved the final draft for publication. AN and RL were responsible for the decision to submit for publication.

## Data sharing statement

All data are publicly available and can be accessed through The DHS Program, https://dhsprogram.com/data/.

## Declaration of interests

Authors declare no competing interests.

## Appendix A Supplementary data

Supplementary Tables A1–A4
