# Supplementary material for: The effect of the COVID-19 pandemic on routine childhood immunization coverage and timeliness in India: retrospective analysis of the National Family Health Survey of 2019–2021 data
Source: Lancet Reg Health Southeast Asia. 2022 Oct 21;8:100099. doi: 10.1016/j.lansea.2022.100099 (PMC9584865; doi:10.1016/j.lansea.2022.100099)
Supplement: Supplementary Tables A1–A4 [file mmc1.docx]

**Supplementary Appendix**

Table A1: Effect of COVID-19 on vaccination receipt

| Model | 1 | 2 | 3 | 4 | 5 | 6 | 7 | 8 | 9 |
| --- | --- | --- | --- | --- | --- | --- | --- | --- | --- |
| Vaccine | BCG | hepb0 | DPT1 | DPT2 | DPT3 | polio1 | polio2 | polio3 | MCV1 |
|  |  |  |  |  |  |  |  |  |  |
| COVID-affected =1 | -0.03** | -0.04** | -0.11** | -0.16** | -0.17** | -0.11** | -0.16** | -0.20** | -0.08** |
|  | 0.01 | 0.02 | 0.01 | 0.01 | 0.02 | 0.01 | 0.01 | 0.02 | 0.03 |
|  |  |  |  |  |  |  |  |  |  |
| Female child | 0 | 0.01 | 0 | 0 | 0 | 0 | 0 | 0 | 0 |
|  | 0.01 | 0.01 | 0.01 | 0.01 | 0.01 | 0.01 | 0.01 | 0.01 | 0.01 |
|  |  |  |  |  |  |  |  |  |  |
| Institutional delivery | 0.06** | 0.20** | 0.01 | 0.05* | 0.06** | 0.02 | 0.04+ | 0.04+ | 0.02 |
|  | 0.01 | 0.02 | 0.02 | 0.02 | 0.02 | 0.02 | 0.02 | 0.02 | 0.03 |
|  |  |  |  |  |  |  |  |  |  |
| *Birth order* |  |  |  |  |  |  |  |  |  |
| Second | 0.02** | 0.04** | 0.04** | 0.05** | 0.05** | 0.02* | 0.04** | 0.04** | 0.03+ |
|  | 0.01 | 0.01 | 0.01 | 0.01 | 0.01 | 0.01 | 0.01 | 0.01 | 0.02 |
| Third | 0.03+ | 0.07** | 0.07** | 0.08** | 0.09** | 0.04* | 0.07** | 0.07** | 0.02 |
|  | 0.02 | 0.02 | 0.02 | 0.02 | 0.02 | 0.02 | 0.02 | 0.02 | 0.03 |
| Fourth or higher | 0.05* | 0.12** | 0.11** | 0.11** | 0.08* | 0.07** | 0.09** | 0.06+ | -0.01 |
|  |  |  |  |  |  |  |  |  |  |
| Observations | 95,731 | 94,842 | 93,515 | 91,993 | 91,024 | 93,744 | 92,027 | 91,183 | 81,727 |
| Pseudo R^2^ | 0.009 | 0.025 | 0.055 | 0.1 | 0.147 | 0.034 | 0.077 | 0.085 | 0.113 |

Note: *HepB0*=Hepatitis B given at birth, *DPT*=Diphtheria, Pertussis, Tetanus, *BCG*=Bacillus Calmette–Guérin, *MCV1*=Measles Conjugative Vaccine, dose 1; Standard errors are below coefficients. +p<0.1, *p<0.05, **p<0.01.

Table A2: Effect of COVID-19 on vaccination receipt within 45 days of eligibility

| Model | 1 | 2 | 3 | 4 | 5 | 6 | 7 | 8 | 9 |
| --- | --- | --- | --- | --- | --- | --- | --- | --- | --- |
| Vaccine | BCG | hepb0 | DPT1 | DPT2 | DPT3 | polio1 | polio2 | polio3 | MCV1 |
|  |  |  |  |  |  |  |  |  |  |
| COVID-affected =1 | 0 | -0.02 | -0.05** | -0.05** | -0.03* | -0.05** | -0.05** | -0.04** | 0.02 |
|  | 0.01 | 0.01 | 0.01 | 0.01 | 0.02 | 0.01 | 0.01 | 0.01 | 0.02 |
|  |  |  |  |  |  |  |  |  |  |
| Female child | 0 | 0 | 0 | 0 | 0.01 | -0.01 | -0.01 | 0 | 0.01 |
|  | 0.01 | 0.01 | 0.01 | 0.01 | 0.01 | 0.01 | 0.01 | 0.01 | 0.01 |
|  |  |  |  |  |  |  |  |  |  |
| Institutional delivery | 0.15** | 0.24** | 0.01 | -0.01 | 0.02 | 0 | 0.01 | 0.03+ | 0.04 |
|  | 0.02 | 0.02 | 0.02 | 0.02 | 0.02 | 0.02 | 0.02 | 0.02 | 0.03 |
|  |  |  |  |  |  |  |  |  |  |
| *Birth order* |  |  |  |  |  |  |  |  |  |
| Second | 0 | 0.01 | 0.01 | 0 | -0.03* | -0.01 | -0.01 | -0.02 | 0.03 |
|  | 0.01 | 0.01 | 0.01 | 0.01 | 0.01 | 0.01 | 0.01 | 0.01 | 0.02 |
| Third | 0 | 0.01 | 0.02 | 0.02 | -0.05* | -0.02 | -0.02 | -0.04 | 0.01 |
|  | 0.02 | 0.02 | 0.02 | 0.02 | 0.02 | 0.02 | 0.02 | 0.02 | 0.03 |
| Fourth or higher | 0.01 | 0.03 | 0.09* | 0.05 | -0.04 | 0.02 | 0.01 | -0.01 | 0.05 |
|  |  |  |  |  |  |  |  |  |  |
| Observations | 113,421 | 115,589 | 107,661 | 105,649 | 104,550 | 108,685 | 108,904 | 110,970 | 82,161 |
| R^2^ | 0.014 | 0.029 | 0.006 | 0.021 | 0.025 | 0.012 | 0.018 | 0.015 | 0.017 |

Note: *HepB0*=Hepatitis B given at birth, *DPT*=Diphtheria, Pertussis, Tetanus, *BCG*=Bacillus Calmette–Guérin, *MCV1*=Measles Conjugative Vaccine, dose 1; Standard errors are below coefficients. +p<0.1, *p<0.05, **p<0.01.

Table A3: Effect of COVID-19 on vaccination receipt within 28 days of eligibility

| Model | 1 | 2 | 3 | 4 | 5 | 6 | 7 | 8 | 9 |
| --- | --- | --- | --- | --- | --- | --- | --- | --- | --- |
| Vaccine | BCG | hepb0 | DPT1 | DPT2 | DPT3 | polio1 | polio2 | polio3 | MCV1 |
|  |  |  |  |  |  |  |  |  |  |
| COVID-affected =1 | 0 | -0.01 | -0.03+ | -0.02 | -0.02+ | -0.03* | -0.04* | -0.03* | 0.02 |
|  | 0.01 | 0.01 | 0.02 | 0.02 | 0.01 | 0.01 | 0.01 | 0.01 | 0.02 |
|  |  |  |  |  |  |  |  |  |  |
| Female child | 0 | 0 | -0.01 | 0 | 0 | -0.01 | -0.01 | 0 | 0.01 |
|  | 0.01 | 0.01 | 0.01 | 0.01 | 0.01 | 0.01 | 0.01 | 0.01 | 0.01 |
|  |  |  |  |  |  |  |  |  |  |
| Institutional delivery | 0.18** | 0.24** | 0.03 | -0.01 | 0.02 | 0.03 | 0.01 | 0.02 | 0 |
|  | 0.02 | 0.02 | 0.02 | 0.02 | 0.02 | 0.02 | 0.02 | 0.02 | 0.03 |
|  |  |  |  |  |  |  |  |  |  |
| *Birth order* |  |  |  |  |  |  |  |  |  |
| Second | 0 | 0.01 | -0.01 | -0.01 | -0.03* | -0.02+ | -0.02+ | -0.02+ | 0 |
|  | 0.01 | 0.01 | 0.01 | 0.01 | 0.01 | 0.01 | 0.01 | 0.01 | 0.02 |
| Third | 0 | 0.01 | -0.01 | 0 | -0.04 | -0.04 | -0.02 | -0.03 | 0.01 |
|  | 0.02 | 0.02 | 0.03 | 0.02 | 0.02 | 0.03 | 0.02 | 0.02 | 0.03 |
| Fourth or higher | 0.03 | 0.02 | 0.03 | 0.04 | -0.03 | -0.01 | 0 | -0.03 | 0.04 |
|  |  |  |  |  |  |  |  |  |  |
| Observations | 113,421 | 115,589 | 107,661 | 105,649 | 104,550 | 108,685 | 108,904 | 110,970 | 82,161 |
| R^2^ | 0.018 | 0.029 | 0.004 | 0.016 | 0.016 | 0.007 | 0.013 | 0.01 | 0.012 |

Note: *HepB0*=Hepatitis B given at birth, *DPT*=Diphtheria, Pertussis, Tetanus, *BCG*=Bacillus Calmette–Guérin, *MCV1*=Measles Conjugative Vaccine, dose 1; Standard errors are below coefficients. +p<0.1, *p<0.05, **p<0.01.

Table A4: Summary results of effect of COVID-19 on vaccination receipt and timely vaccination, subsample analysis

|  |  | Receipt of vaccination | | Receipt of vaccination within 45 days of eligibility | |
| --- | --- | --- | --- | --- | --- |
| Vaccine | Subsample | Coefficient | Sample size | Coefficient | Sample size |
| BCG | Urban | -0.03** (0.01) | 76,345 | 0 (0.012) | 90,692 |
| BCG | Rural | -0.05* (0.02) | 19,386 | -0.01 (0.025) | 22,729 |
| BCG | Poor | -0.03* (0.012) | 47,941 | 0.01 (0.015) | 55,756 |
| BCG | Rich | -0.04** (0.013) | 47,790 | -0.01 (0.016) | 57,665 |
| BCG | Male | -0.04* (0.017) | 46,377 | 0.03 (0.022) | 54,926 |
| BCG | Female | -0.04* (0.018) | 49354 | 0.01 (0.024) | 58,495 |
| hepb0 | Urban | -0.04* (0.017) | 75630 | -0.02 (0.013) | 92,487 |
| hepb0 | Rural | -0.06 (0.038) | 19212 | 0.01 (0.028) | 23,102 |
| hepb0 | Poor | -0.03+ (0.02) | 47401 | -0.01 (0.015) | 57,360 |
| hepb0 | Rich | -0.06** (0.024) | 47441 | -0.01 (0.018) | 58,229 |
| hepb0 | Male | -0.07* (0.03) | 45938 | -0.01 (0.022) | 55,917 |
| hepb0 | Female | -0.08* (0.031) | 48904 | -0.01 (0.024) | 59,672 |
| DPT1 | Urban | -0.11** (0.013) | 74519 | -0.02 (0.017) | 85,784 |
| DPT1 | Rural | -0.14** (0.028) | 18996 | -0.07* (0.036) | 21,877 |
| DPT1 | Poor | -0.12** (0.016) | 46744 | 0 (0.021) | 52,567 |
| DPT1 | Rich | -0.11** (0.018) | 46771 | -0.06** (0.023) | 55,094 |
| DPT1 | Male | -0.13** (0.023) | 45254 | -0.04 (0.031) | 52,015 |
| DPT1 | Female | -0.1** (0.025) | 48261 | -0.01 (0.032) | 55,646 |
| DPT2 | Urban | -0.15** (0.015) | 73282 | -0.02 (0.017) | 84,171 |
| DPT2 | Rural | -0.2** (0.034) | 18711 | -0.04 (0.036) | 21,478 |
| DPT2 | Poor | -0.17** (0.019) | 45952 | -0.01 (0.02) | 51,659 |
| DPT2 | Rich | -0.15** (0.02) | 46041 | -0.04 (0.023) | 53,990 |
| DPT2 | Male | -0.18** (0.027) | 44519 | -0.05+ (0.03) | 51,040 |
| DPT2 | Female | -0.11** (0.029) | 47474 | -0.03 (0.032) | 54,609 |
| DPT3 | Urban | -0.16** (0.018) | 72488 | -0.02 (0.015) | 83,269 |
| DPT3 | Rural | -0.22** (0.039) | 18536 | -0.06+ (0.034) | 21,281 |
| DPT3 | Poor | -0.19** (0.022) | 45526 | -0.01 (0.018) | 51,372 |
| DPT3 | Rich | -0.15** (0.024) | 45498 | -0.04+ (0.022) | 53,178 |
| DPT3 | Male | -0.19** (0.031) | 44049 | -0.06* (0.027) | 50,521 |
| DPT3 | Female | -0.14** (0.034) | 46975 | -0.02 (0.029) | 54,029 |
| polio1 | Urban | -0.1** (0.013) | 74710 | -0.03+ (0.017) | 86,725 |
| polio1 | Rural | -0.11** (0.028) | 19034 | -0.04 (0.035) | 21,960 |
| polio1 | Poor | -0.12** (0.015) | 46891 | -0.01 (0.021) | 53,141 |
| polio1 | Rich | -0.09** (0.017) | 46853 | -0.05* (0.022) | 55,544 |
| polio1 | Male | -0.11** (0.023) | 45376 | -0.05 (0.03) | 52,516 |
| polio1 | Female | -0.08** (0.024) | 48368 | 0.02 (0.031) | 56,169 |
| polio2 | Urban | -0.16** (0.016) | 73329 | -0.03* (0.016) | 86,886 |
| polio2 | Rural | -0.2** (0.035) | 18698 | -0.06+ (0.035) | 22,018 |
| polio2 | Poor | -0.19** (0.019) | 46020 | -0.02 (0.02) | 53,490 |
| polio2 | Rich | -0.13** (0.021) | 46007 | -0.06** (0.022) | 55,414 |
| polio2 | Male | -0.18** (0.028) | 44555 | -0.07* (0.029) | 52,675 |
| polio2 | Female | -0.09** (0.03) | 47472 | -0.02 (0.031) | 56,229 |
| polio3 | Urban | -0.18** (0.019) | 72629 | -0.02+ (0.014) | 88,457 |
| polio3 | Rural | -0.29** (0.043) | 18554 | -0.06* (0.031) | 22,513 |
| polio3 | Poor | -0.21** (0.023) | 45636 | -0.02 (0.017) | 54,821 |
| polio3 | Rich | -0.18** (0.026) | 45547 | -0.04* (0.02) | 56,149 |
| polio3 | Male | -0.21** (0.034) | 44131 | -0.08** (0.026) | 53,662 |
| polio3 | Female | -0.16** (0.037) | 47052 | -0.02 (0.027) | 57,308 |
| MCV1 | Urban | -0.09** (0.03) | 64983 | 0.02 (0.024) | 65,336 |
| MCV1 | Rural | -0.06 (0.066) | 16744 | 0.01 (0.058) | 16,825 |
| MCV1 | Poor | -0.11** (0.036) | 40745 | 0.03 (0.031) | 40,489 |
| MCV1 | Rich | -0.06 (0.04) | 40982 | 0.01 (0.033) | 41,672 |
| MCV1 | Male | -0.1+ (0.053) | 39464 | -0.02 (0.045) | 39,643 |
| MCV1 | Female | -0.11+ (0.058) | 42263 | 0.05 (0.047) | 42,518 |

Note: *HepB0*=Hepatitis B given at birth, *DPT*=Diphtheria, Pertussis, Tetanus, *BCG*=Bacillus Calmette–Guérin, *MCV1*=Measles Conjugative Vaccine, dose 1; Standard errors are below coefficients. +p<0.1, *p<0.05, **p<0.01.
